# Soil bacterial diversity is positively associated with air temperature in the maritime Antarctic

**DOI:** 10.1038/s41598-019-39521-7

**Published:** 2019-02-25

**Authors:** Paul G. Dennis, Kevin K. Newsham, Steven P. Rushton, Anthony G. O’Donnell, David W. Hopkins

**Affiliations:** 1 School of Earth and Environmental Sciences, The University of Queensland, Brisbane, QLD 4072 Australia; 2 NERC British Antarctic Survey, Madingley Road, Cambridge, CB3 0ET UK; 3 School of Natural and Environmental Sciences, Newcastle University, Newcastle upon Tyne, NE1 7RU UK; 4 University of Western Australia, 35 Stirling Highway, Crawley, WA 6009 Australia; 5 Scotland’s Rural College, Peter Wilson Building, West Mains Road, Edinburgh, EH9 3JG UK

## Abstract

Terrestrial ecosystems in the maritime Antarctic experienced rapid warming during the latter half of the 20^th^ century. While warming ceased at the turn of the millennium, significant increases in air temperature are expected later this century, with predicted positive effects on soil fungal diversity, plant growth and ecosystem productivity. Here, by sequencing 16S ribosomal RNA genes in 40 soils sampled from along a 1,650 km climatic gradient through the maritime Antarctic, we determine whether rising air temperatures might similarly influence the diversity of soil bacteria. Of 22 environmental factors, mean annual surface air temperature was the strongest and most consistent predictor of soil bacterial diversity. Significant, but weaker, associations between bacterial diversity and soil moisture content, C:N ratio, and Ca, Mg, PO_4_^3−^ and dissolved organic C concentrations were also detected. These findings indicate that further rises in air temperature in the maritime Antarctic may enhance terrestrial ecosystem productivity through positive effects on soil bacterial diversity.

## Introduction

During the latter half of the 20th century, air temperatures in the maritime Antarctic rose more rapidly than in any other region of the Southern Hemisphere (c. 0.2 °C increases in mean near surface annual temperature per decade)^1^ and then stabilised before the turn of the millennium^2^. Warming in the region led to the collapse of ice shelves, the retreat of glaciers, and increases in the frequencies of precipitation^1,3^, the growth of bryophytes^4^, the occurrence of invasive species^5^ and the ranges of native plants^6,7^. Much less is known, however, about how warming and its associated environmental changes might influence the diversity of soil micro-organisms (i.e., bacteria, archaea and microeukarya) across the region. This knowledge gap is significant, as changes to microbial diversity may affect terrestrial ecosystem functioning due to the important roles of many taxa as autotrophs, saprotrophs, pathogens and symbionts. In addition, climate models, assuming only moderate anthropogenic greenhouse gas emissions, predict that in the latter half of this century the maritime Antarctic will warm at similar rates to those observed between the 1950s and late 1990s^8^.

Soil fungal diversity in the region is positively associated with air temperature, with warming-induced changes in fungal community composition being predicted to enhance ecosystem productivity through effects on nutrient cycling^9^. At present, however, little is known of how further climate warming will influence the diversity of bacteria in maritime Antarctic soils. Current knowledge about the regional-scale factors influencing soil bacterial diversity in maritime Antarctica derives predominantly from a survey conducted between 2003 and 2005^10–15^. Sanger sequencing, microarray analyses and denaturing gradient gel electrophoresis of bacterial 16S rRNA genes in soils from this survey indicate that alpha diversity decreases, and community composition changes, with increasing latitude^10,11,15^. However, as soil was sampled from five to eight locations between the Falkland Islands and the Ellsworth Mountains in the continental Antarctic, with temperature being recorded at three sites^10–15^, the environmental drivers of this spatial pattern in bacterial diversity remain unclear.

**Figure 1:**
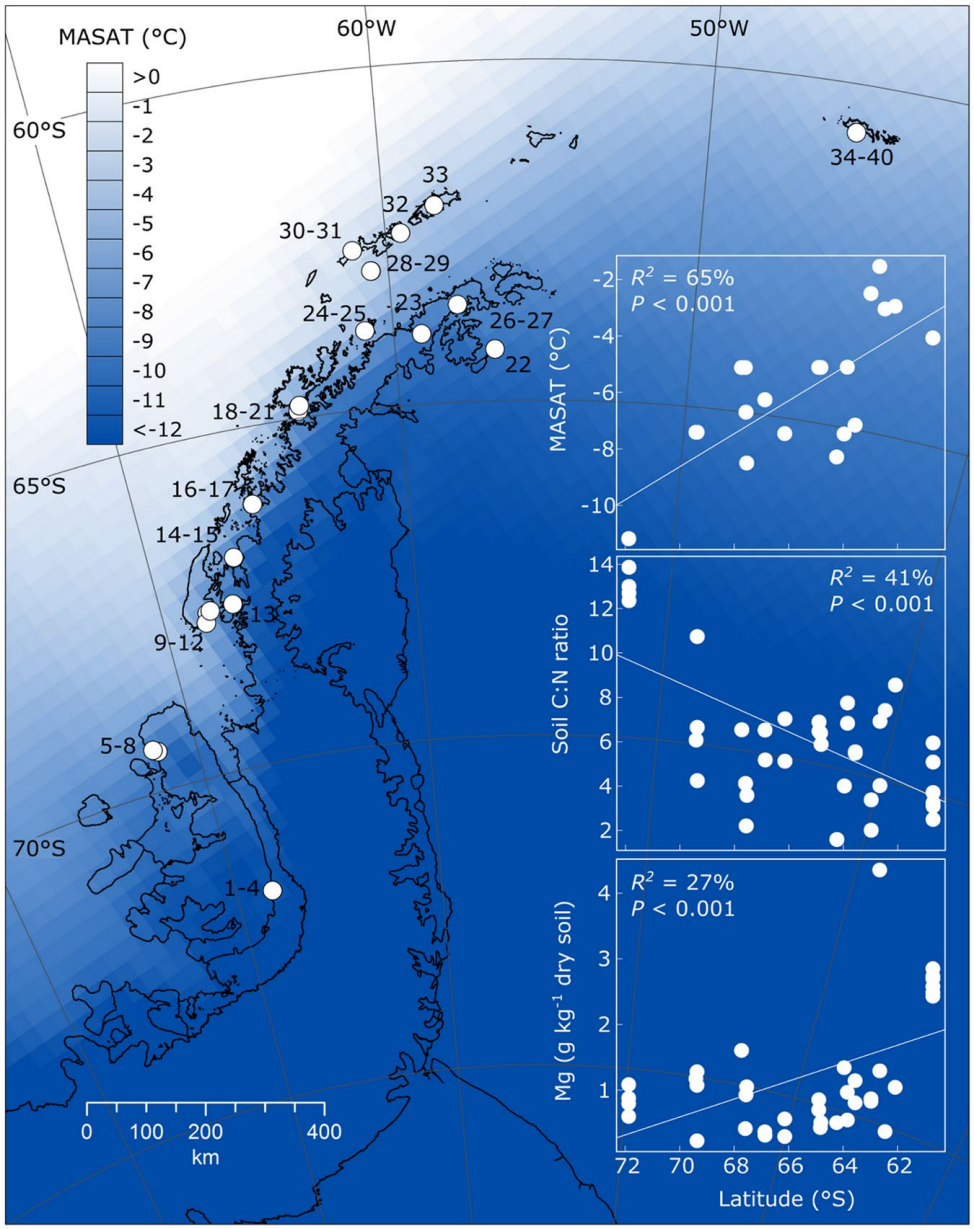
Locations of sampling sites along the climatic gradient. Site names, latitudes, longitudes, mean annual surface air temperatures (MASAT), altitudes and soil pH values are shown in Supplementary Table S1. MASAT for 2007 are shown as a colour gradient. Upper, middle and lower insets show MASAT, soil C:N ratio and Mg concentration as functions of latitude, respectively. The image was generated using ArcGIS v. 10.3^63^.

Here, we test the hypothesis that the diversity of maritime Antarctic soil bacteria is significantly associated with reductions in temperature, liquid water and nutrient availability at higher latitudes^10^. We studied bacterial diversity in 40 soils sampled during the 2007–2008 austral spring and summer from along a 1,650 km climatic gradient encompassing almost the entire maritime Antarctic (Fig. 1, Supplementary Table S1). The soils that were sampled were devoid of vegetation, and are hence typical of maritime Antarctic terrestrial ecosystems. For each sample, 22 environmental factors were measured, including soil pH, electrical conductivity, moisture content, ion and element concentrations and mean annual surface air temperature (MASAT), which was derived from the Regional Atmospheric Climate Model^16^. The diversity of soil bacterial communities was characterised using high-throughput phylogenetic marker gene sequencing. Significant associations between environmental factors and the diversity and composition of bacterial communities in the region were then identified.

## Results

### Associations between latitude and environmental factors

We observed a significant increase (*r*^2^ = 65%, *P* < 0.001) in MASAT between southeast Alexander Island at 72°S (MASAT −11 °C) and Signy Island at 60 °S (MASAT −4 °C), with a 0.58 °C increase in air temperature for each degree decrease in latitude (Fig. 1; Supplementary Table S2). In addition, we found that the ratio of soil organic C to N decreased at lower latitudes (*r*^2^ = 41%, *P* < 0.001; Fig. 1; Supplementary Table S2), which was principally owing to the highest C:N ratios being recorded in four soils from the most southerly location along the gradient (Fig. 1). An association was also found between latitude and soil Mg concentration, with higher concentrations of the element in soils from lower latitudes (*r*^2^ = 27%, *P* < 0.001; Fig. 1; Supplementary Table S2). None of the other environmental factors were significantly associated with latitude (Supplementary Table S2).

### Factors influencing soil bacterial alpha diversity

**Figure 2:**
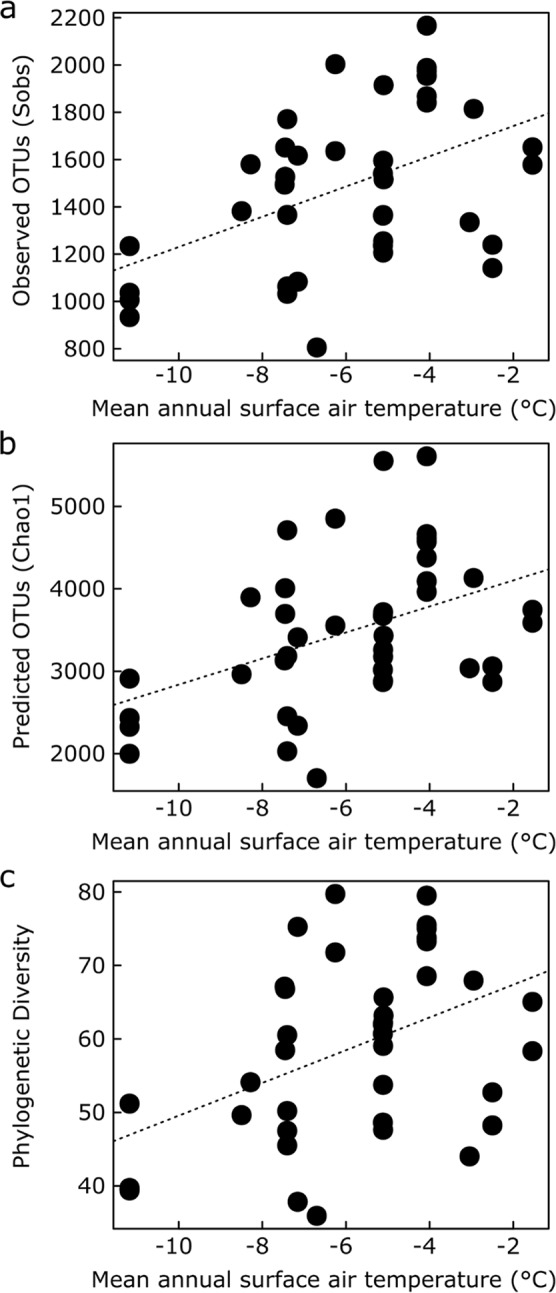
(**a**) Observed numbers of OTUs, (**b**) predicted (Chao 1) numbers of OTUs and (**c**) Faith’s Phylogenetic Diversity Index values as functions of mean annual surface air temperature along the climatic gradient. The fitted lines are from univariate regression analyses.

Stepwise multiple regression modelling indicated that MASAT was the strongest and most consistent predictor of the numbers of observed and predicted (Chao1) bacterial taxa, as well as the phylogenetic diversity of each community (Table 1; Fig. 2). For every degree Celsius increase in MASAT, stepwise multiple regression models indicated an additional 32.8 observed taxa, 136.8 predicted taxa and a 1.3 unit increase in Faith’s Phylogenetic Diversity values (Table 1). All three measures of soil bacterial alpha diversity were also positively, but less closely, associated with soil moisture concentration (Table 1). Lastly, the number of observed OTUs was positively associated with Mg concentration, and the two other measures of alpha diversity were negatively associated with concentrations of water extractable PO_4_^3−^, or dissolved organic C and Ca (Table 1). Soil pH value, which ranged from pH 5.1–7.9 (Supplementary Table S1), was not found to influence bacterial alpha diversity.

**Table 1:** Significant predictors of soil bacterial alpha diversity derived from stepwise multiple regression models.

Response variable	*r*^2^ (%)	Predictor variable	Slope	*F* value	*P* value
Observed OTUs	44.0	MASAT*	32.83	14.05	<0.001
		Moisture concentration	7.95	5.53	0.024
		Mg concentration	1.57 × 10^−2^	8.71	0.006
Predicted OTUs (Chao 1)	35.8	MASAT*	136.80	10.51	0.003
		Moisture concentration	30.16	4.57	0.039
		Water extractable PO_4_^3−^ concentration	−13.42	4.95	0.032
Phylogenetic Diversity	54.2	MASAT*	1.26	16.7	<0.001
		Moisture concentration	0.95	9.26	0.004
		Dissolved organic C concentration	−3.91	14.14	<0.001
		Ca concentration	−1.07 × 10^−4^	7.76	0.009

*Mean annual surface air temperature. MASAT was expressed in degrees Celsius, moisture concentrations as percentages and Mg, PO_4_^3−^, C and Ca concentrations in mg kg^−1^ in these analyses, respectively. Error degrees of freedom were 35 (observed and predicted nos OTUs) and 36 (Phylogenetic Diversity).

### Factors influencing soil bacterial beta diversity

Stepwise PERMANOVA analyses indicated that MASAT was the strongest predictor of differences in bacterial community composition between soils (Table 2). Significant associations were also detected between changes in soil bacterial community composition and the concentrations of Mg, moisture, dissolved organic C and soil C:N ratio (Table 2). Soil pH had no influence on beta diversity.

**Table 2:** Data from a stepwise PERMANOVA model showing significant predictors of soil bacterial beta diversity.

Predictor variable	*r*^2^ (%)	*F* value	*P* value
MASAT*	2.77	6.33	<0.001
Mg concentration	2.08	4.75	<0.001
Moisture concentration	1.99	4.54	<0.001
Dissolved organic C concentration	1.49	3.41	0.016
C:N ratio	1.45	3.32	0.026

*Mean annual surface air temperature. MASAT was expressed in degrees. Celsius, Mg and dissolved organic C concentrations in mg kg^−1^ and moisture concentrations as percentages, respectively. C:N ratio was unitless. Error degrees of freedom were 34.

**Figure 3:**
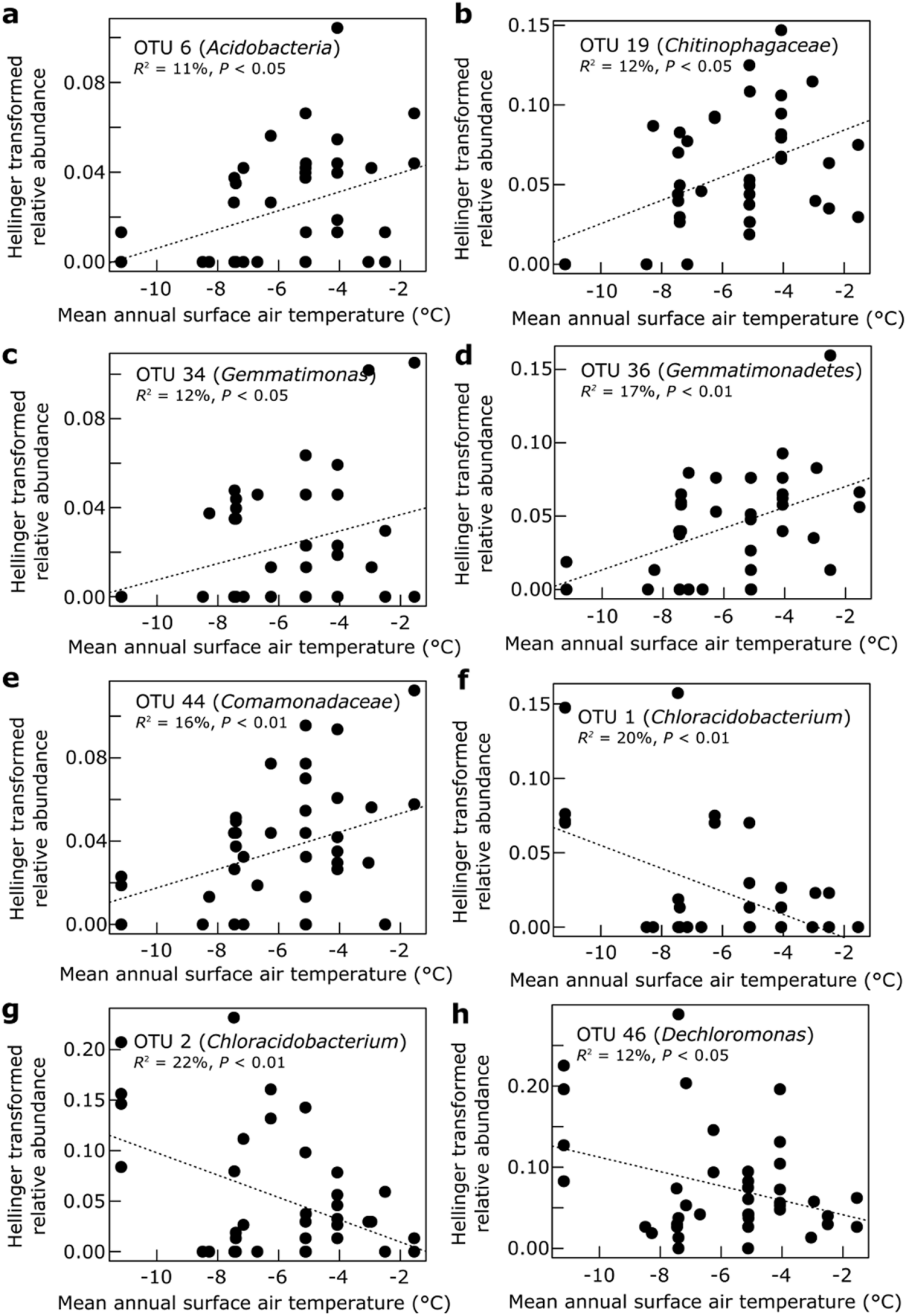
(**a**–**h**) The relative abundances of eight OTUs as functions of mean annual surface air temperature along the climatic gradient.

**Figure 4:**
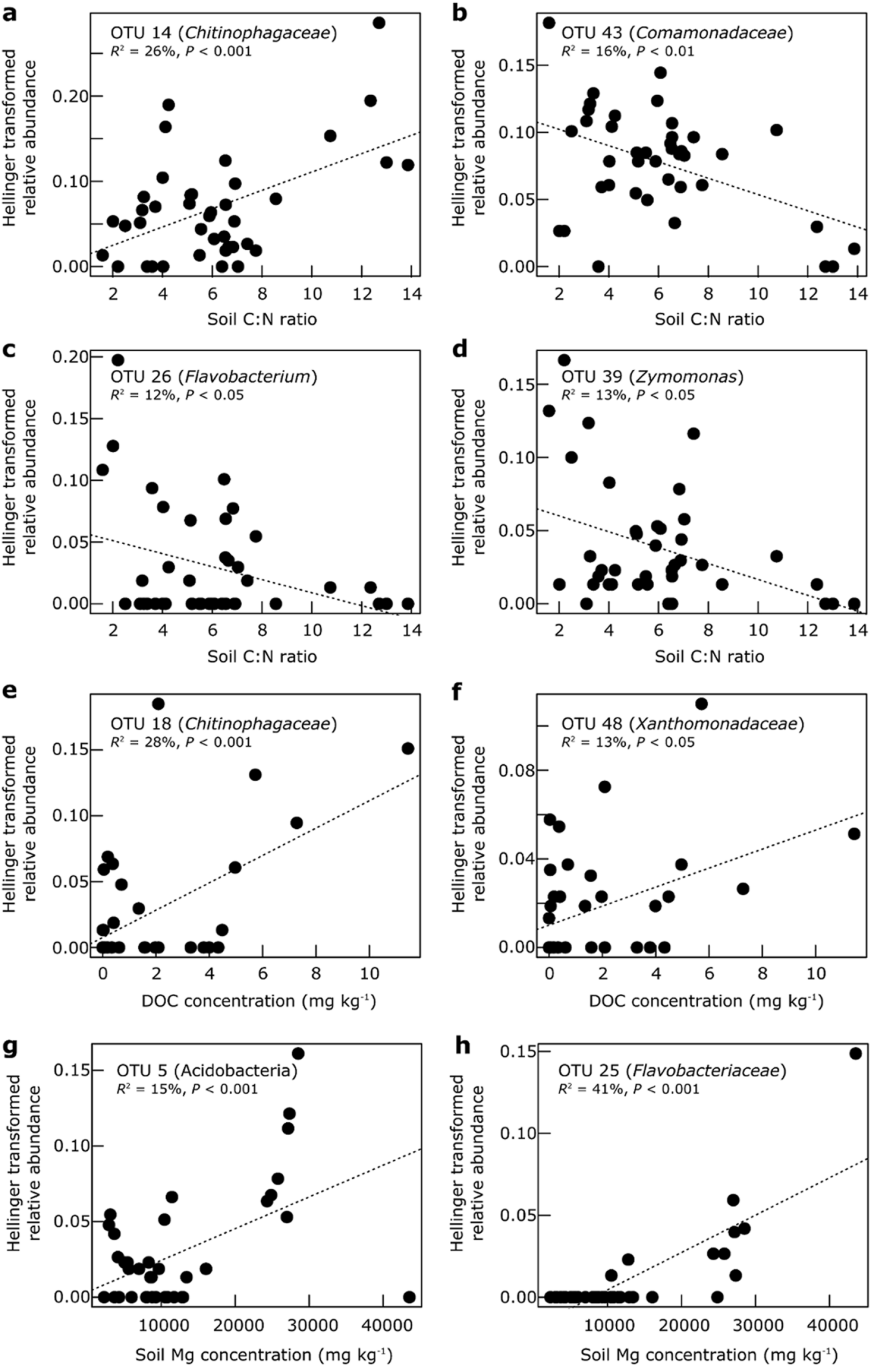
The relative abundances of eight OTUs as functions of (**a**–**d**) soil C:N ratio, (**e**,**f**) dissolved organic C concentration and (**g**,**h**) Mg concentration.

Univariate regression analyses showed that the relative abundances of members of the *Acidobacteria* (OTU 6), *Chitinophagaceae* (OTU 19, *Bacteroidetes*), *Gemmatimonadetes* (OTUs 34 and 36) and *Comamonadaceae* (OTU 44, *Betaproteobacteria*) were positively associated with MASAT (Fig. 3a–e), and that members of the *Chloracidobacterium* (OTUs 1 and 2, *Acidobacteria*) and *Dechloromonas* (OTU 46, *Betaproteobacteria*) were negatively associated with MASAT (Fig. 3f–h). The relative abundances of four bacterial taxa were found to be related to soil C:N ratio, with a member of the *Chitinophagaceae* (OTU 14) being positively associated with C:N ratio (Fig. 4a), and a *Comamonadaceae* (OTU 43), a *Flavobacterium* (OTU 26, *Bacteroidetes*) and a *Zymomonas* (OTU 39, *Alphaproteobacteria*) population being negatively associated with C:N ratio (Fig. 4b–d). Similarly, another member of the *Chitinophagaceae* (OTU 18) and a representative of the *Xanthomonadaceae* (OTU 48, *Gammaproteobacteria*) were found to be positively associated with dissolved organic C concentration (Fig. 4e,f). Members of the *Acidobacteria* (OTU 5) and the *Flavobacteriaceae* (OTU 25, *Bacteroidetes*) were relatively more abundant in soils with high Mg concentrations (Fig. 4g,h).

### Soil bacterial community composition

**Figure 5:**
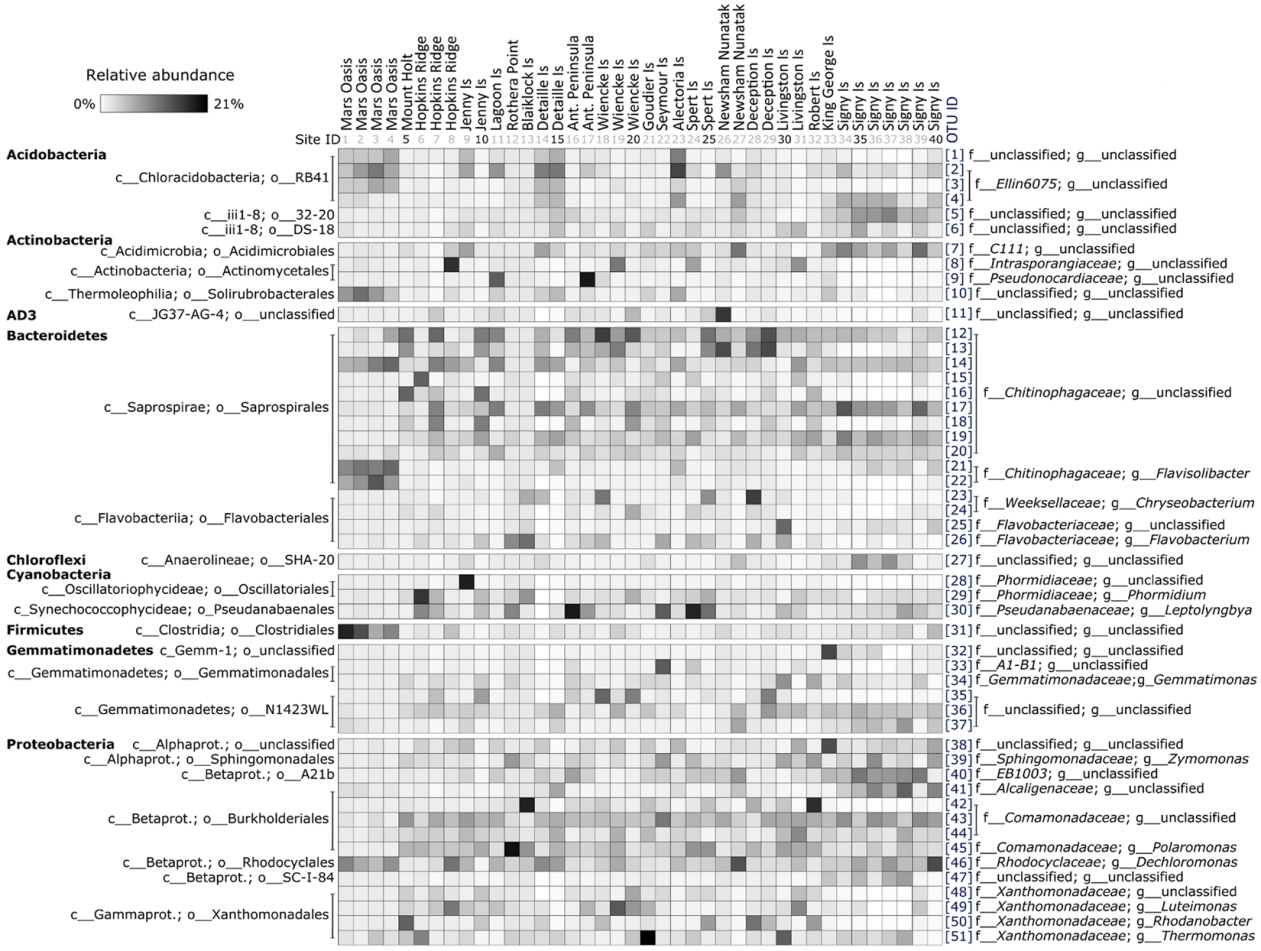
Heatmap illustrating the abundances of the dominant (>5%) bacterial taxa recorded in each soil as well as those that were significantly associated with the predictor variables shown in Table 2.

In addition to members of the *Acidobacteria*, *Bacteroidetes*, *Gemmatimonadetes* and *Proteobacteria*, representatives of the *Actinobacteria* and *Cyanobacteria* were abundant in maritime Antarctic soils (Fig. 5). Those of the Candidate phylum AD3, *Chloroflexi* and *Firmicutes* were also abundant in soils at several locations but were more sporadically distributed (Fig. 5). The bacterial taxa detected were closely related to those observed in other surveys of soils from Antarctica or other cold and arid environments (Supplementary Figs S1–S5).

## Discussion

Our analyses, based on 40 soils sampled from along a 1,650 km climatic gradient, indicate that air temperature is the strongest and most consistent factor explaining latitudinal changes in maritime Antarctic soil bacterial diversity. In accordance with other studies in the region showing positive associations between air temperature and soil fungal diversity^9^, decreased bryophyte and lichen diversity in more southerly habitats^17,18^ and increased plant growth rates in response to warming in the latter half of the 20th century^4,6^, the data reported here indicate that the warming which is predicted to occur towards the end of the current century under moderate greenhouse gas emission scenarios^8^ is likely to lead to increased diversity of soil bacterial communities across the region. These predicted changes to soil bacterial diversity are likely to be effected by bird^19^ and human vectors^5^, as well as by wind currents, which are thought to be capable of transporting bryophyte spores, pollen and microbes from South America to the maritime Antarctic, and southwards along the Antarctic Peninsula^20–22^.

Although bacterial diversity is probably more closely associated with soil temperature than air temperature, the inaccessible nature of the majority of the sites studied here – some of which had not previously been visited – precluded the long-term measurement of soil temperatures and their inclusion in regression models as predictors for bacterial diversity. However, previous research indicates that, despite air and soil temperatures becoming decoupled during winter in maritime Antarctica owing to the insulating effects of snow and ice cover^23^, air temperature is the best predictor for soil temperature in the region^24^ (Supplementary Fig. S6). Rising air and soil temperatures might also affect the frequency of freeze-thaw cycles at soil surfaces, which short-term laboratory studies have found to have marginal effects on maritime Antarctic bacterial community composition^12^. However, rises in temperature will increase the availability of liquid water, a key driver of biological diversity in Antarctic terrestrial ecosystems^25^. Indeed, in this study, soil moisture was positively associated with the number of bacterial taxa in soil and was a significant predictor of changes to the relative frequencies of taxa between soils. In these frigid and usually arid habitats, growth is typically limited to periods between late spring and late summer, when soil temperatures rise above 0 °C and liquid water becomes available^23,26^, with the increased number of bacterial taxa in warmer and wetter soils likely reflecting the enhanced potential for metabolic activity for a wider range of taxa during late austral spring and summer^9,18^. Although rising air temperatures across the region might lead to accelerated soil thaw and increased meltwater and summertime precipitation^27^, amplifying the effects of climate warming on soil bacterial diversity, we cannot discount the possibility that changing atmospheric circulation patterns, such as increased frequencies of easterly winds bringing cold, dry air from continental Antarctica^28^, will force the drying of maritime Antarctic soils, limiting the effects of rising air temperatures on soil bacterial diversity.

Increased soil bacterial diversity associated with rising air temperatures can be expected to enhance terrestrial ecosystem productivity by accelerating the mineralisation rates of C and N and other elements in soil that limit the growth of other organisms, in particular higher plants and bryophytes^12,13,29^. The analyses here suggest that future rises in air temperature in the region will result in increases in the abundances of specific bacterial taxa, notably members of the *Acidobacteria* – a frequent phylum in more northerly maritime Antarctic soils^10^ – and the *Gemmatimonadetes*, *Chitinophagaceae* and *Comamonadaceae*. Increases in the abundances of these taxa might be expected to enhance the decomposition of chitin and other soil organic compounds^14,30^ and, given that a relative of OTU 34, *Gemmatimonas* sp. AP64, is capable of photosynthesis^31^, perhaps also to increase the fixation of atmospheric C into soil. However, of the three taxa that were found here to decrease in abundance in warmer soils, two (OTUs 1 and 2, both *Chloracidobacterium*) are close relatives of the photoheterotroph *Candidatus* Chloracidobacterium thermophilum^32^, suggesting decreased C fixation into warmer maritime Antarctic soils. Reductions in these taxa may hence potentially counteract any changes arising from increases in the abundances of members of the *Gemmatimonadetes*, representing a shift in the structure of the microbial photoautotroph community.

Increasing air temperatures in maritime Antarctica are thought not only to enhance plant growth and soil fungal diversity^4,6,9^, but also to accelerate mineralisation rates and soil nutrient cycling^33^. Maritime Antarctic soils in which there are greater availabilities of inorganic nutrients might be expected to support more active^34^ and perhaps more diverse bacterial communities. This expectation was only weakly supported by the multiple regression analyses here, which indicated a positive effect of soil Mg concentration on the observed number of bacterial taxa, with univariate regressions also indicating increased abundances of members of the *Acidobacteria* and *Flavobacteriaceae* in soils containing higher concentrations of the element. In contrast, the multiple regressions indicated that the concentrations of dissolved organic C, water extractable PO_4_^3−^ and Ca were all negatively associated with the predicted numbers of soil bacterial species and phylogenetic diversity, suggesting that the positive effects of warming on maritime Antarctic soil bacterial diversity may be counteracted by increased concentrations of soil nutrients associated with more productive soil and plant communities^33^. These findings corroborate observations from the Arctic, showing reductions in bacterial diversity and evenness in nutrient-amended soils associated with increased relative abundances of copiotrophic *Alphaproteobacteria* and *Betaproteobacteria*^35,36^. The substantial decline reported here in phylogenetic diversity associated with increasing dissolved organic C concentrations in soil is likely to have arisen, at least in part, from increases in the abundances of specific taxa such as members of the *Bacteroidetes* and *Gammaproteobacteria*, close relatives of which exhibit copiotrophic behaviour^29^. Univariate regressions also indicated that soils with low C:N ratios, which form in warmer environments owing to faster organic matter decomposition^37^, and may be indicative of nutrient-rich habitats, were preferred by *Zymomonas*, *Flavobacterium* and a member of the *Comamonadaceae*, representatives of phyla frequent in soils with high rates of C turnover^29^.

In agreement with previous studies in the maritime Antarctic that have found no influence of soil pH on soil bacterial alpha diversity or community composition^10,15^, or in which pH has been identified as an only marginally significant predictor (*P* > 0.06) for the frequencies of C and N cycling genes^14^, soil pH had no influence on bacterial alpha or beta diversity in the present study. This is in stark contrast to continental scale studies across North America and South America, which report highly significant first order polynomial relationships between soil pH and bacterial diversity, and no effects of mean annual temperature on diversity, in 88–98 soils sampled from Argentina through to northern Alaska^38,39^. This disparity is probably due to the absence from the analyses here of strongly acidic soils, in which there are highly significant linear increases in bacterial diversity between pH 3.5–5.0 in the Americas^38,39^. In contrast, no changes in bacterial diversity are recorded in North American and South American soils over the pH range of the soils studied here (pH 5.1–7.9)^38,39^. Nevertheless, why temperature influences soil bacterial diversity in maritime Antarctica and not in the Americas remains to be resolved. One possible explanation is that the much harsher environmental conditions encountered in maritime Antarctic soils, such as desiccation, low temperatures (<0 °C for approximately eight months each year and annual minima of between −15 °C and −40 °C) and wide temperature fluctuations (annual ranges 35–65 °C)^23^ exert stronger selection pressures on bacterial taxa than in the soils sampled from across South America and North America, of which only six are exposed to annual mean temperatures of <0 °C^38,39^. This view is supported by the finding here that the taxa of bacteria present in maritime Antarctic soils – which are phylogenetically similar to those previously recorded in the soils of the region^10,40–42^ – have affinities with taxa encountered in other cold and arid environments, such as ice from the McMurdo Dry Valleys in Continental Antarctica^43^, a Lake Vostok ice core^44^ and hyper-arid soils from the Chilean Andes^45^.

## Conclusions

The data reported here indicate that temperature is the predominant factor determining the diversity of bacterial communities in maritime Antarctic soils. Future rises in air temperature, predicted to occur later this century under moderate greenhouse gas emission scenarios^8^, are thus likely to lead to increased numbers of bacterial species in the soils of the region, enhancing terrestrial ecosystem productivity. However, increased concentrations of soil nutrients such as PO_4_^3−^, Ca and dissolved organic C, which are negatively associated with bacterial diversity, may counteract the positive effects of rising air temperatures on the numbers of bacterial species in soil.

## Methods

### Soil sampling

Soils without plant cover were sampled from between Signy Island (60°S) in the South Orkney Islands and Alexander Island (72°S) in the southern maritime Antarctic in the 2007–2008 austral spring and summer. The uppermost *c*. 50 mm of soil was collected in DNA/RNAase treated plastic tubes from each of five locations at each site and was bulked. Soils were immediately snap frozen by immersion in a mixture of dry ice and ethanol (*c*. −80 °C) and were maintained at this temperature until they were processed.

### Soil physicochemical characteristics

Analyses of the concentrations of soil moisture, elements and water-extractable ions were performed on 4 mm-sieved soils, as described previously^9^.

### Air temperature data

Mean annual surface air temperature (MASAT) data for each site, gridded at a horizontal resolution of 55 × 55 km for the year 2007, were derived from the Regional Atmospheric Climate Model^16^. Although long-term soil temperature measurements for the majority of the sites studied here are unavailable, an analysis of five years of air and soil temperatures at Mars Oasis, the southernmost site sampled, shows that there is a close correlation between daily mean air temperature measured at 1 m above ground level and soil surface temperatures at 0–5 cm depth (Supplementary Fig. S6).

### DNA extraction, PCR amplification and 454 pyrosequencing

Total DNA was extracted from soils as previously described^9^. PCRs were performed on 2 µl DNA extracts, in 1× High Fidelity PCR Buffer (Invitrogen), with 100 nM of each dNTP (Invitrogen), 2 mM MgSO_4_ (Invitrogen), 1 unit of Platinum^®^ Taq High Fidelity (Invitrogen), and 400 nM of each universal bacterial primer, made up to 30 µl total volume with molecular biology grade water. The forward primer was 27F (5′ AGAGTTTGATCCTGGCTCAG), 5′-labelled with the 454 FLX sequencing primer adapter B sequence. The reverse primer was 338R (5′ TGCTGCCTCCCGTAGGAGT), 5′-labelled with a sample specific barcode sequence^46^ and the 454 FLX sequencing primer adapter A sequence. Thermocycling conditions were as follows: 94 °C for 2 min; five cycles touchdown at 94 °C for 30 s, 60–56 °C for 30 s, 68 °C for 45 s, and then 30 cycles of 94 °C for 30 s, 55 °C for 30 s, 68 °C for 30 s and a final extension step at 68 °C for 8 min. Amplicons were purified and normalised to 25 ng DNA per sample using a SequalPrep^TM^ Normalization Plate Kit according to the manufacturer’s instructions (ThermoFisher Scientific). Amplicons were then pooled for 454 GS-FLX Titanium pyrosequencing (Roche), which was performed at the NERC Biomolecular Analysis Facility (University of Liverpool, UK).

### Processing of sequence data

Sequences were quality filtered and dereplicated using the QIIME script split_libraries.py with the homopolymer filter deactivated^47^ and then checked for chimeras against the October 2013 release of the GreenGenes database using UCHIME^48^ v. 3.0.617. Homopolymer errors were corrected using Acacia^49^ v. 1.48. Using QIIME, sequences were then clustered at 97% similarity using UCLUST^50^ and cluster representatives were randomly selected. GreenGenes taxonomy^51^ (October 2013 release) was then assigned to the cluster representatives using BLAST, and tables with the abundances of each Operational Taxonomic Unit (OTU) and its taxonomic assignment in each sample were generated. In addition, full length sequences that were identified as the nearest BLAST matches for each OTU were aligned and a midpoint rooted phylogenetic tree was generated.

A plot of the observed number of taxa relative to the number of sequences per sample showed that the sequencing did not account for all of the taxa present in the soils (Supplementary Fig. S7). Rarefaction^52^ was hence used to calculate the expected number of taxa for an equal number of sequences per sample. The numbers of reads were rarefied to the nearest multiple of 50 sequences below the minimum number of sequences per sample (5,734) by re-sampling the OTU table. All comparisons of diversity were subsequently based on rarefied datasets comprising 5,700 sequences per sample. Inspection of the 95% confidence intervals for each rarefied sample (Supplementary Fig. S7) showed that there was considerably more between-than within-sample variation, indicating that the mean alpha diversity values used in subsequent analyses adequately represented the within-sample diversity to facilitate robust comparisons between samples. The mean number of observed OTUs, predicted OTUs (Chao1)^53^ and Faith’s Phylogenetic Diversity Index values^54^ were calculated using QIIME.

### Statistical analyses

Relationships between latitude, mean annual surface air temperature (MASAT), altitude and the soil physicochemical characteristics were identified using Pearson’s correlations. The influence of MASAT and the soil physicochemical factors on the alpha diversity metrics (i.e., the numbers of observed and predicted [Chao1] OTUs, and Faith’s Phylogenetic Diversity Index values) were assessed using stepwise multiple regression analyses. The influence of MASAT and the soil physicochemical parameters on the composition of bacterial communities (beta diversity) was assessed using Permutational Multivariate Analysis of Variance^55^ (PERMANOVA) as implemented in the Vegan R package^56^. Parsimonious PERMANOVA models were built by forward selection of significant predictors and the OTU relative abundances were Hellinger transformed prior to analysis. All analyses were implemented using R version 3.2.3^57^.

### Phylogeographical analyses

Bacterial 16S rRNA gene amplicon sequences from the 2003–2005 survey of maritime Antarctic soils^10^ (Genbank ACC: EF219488 – EF221599) were downloaded and aligned with the GreenGenes database (October 2013 release) and the most dominant OTUs from the present study using PyNAST^58^. All sequences were then inserted into the full GreenGenes phylogeny (October 2013 release) by maximum parsimony using Arb^59^. Sequences neighbouring the OTU sequences from this study were selected and then aligned in MEGA6^60^ using MUSCLE^61^. Phylogenetic trees were inferred in MEGA6 using the maximum likelihood method based on the Jukes-Cantor model^62^.

## Supplementary information

Supplementary Information

## Data Availability

The 16S rRNA gene amplicon sequences associated with this study have been deposited in the NCBI SRA under accession: PRJNA213362.
