# Supplementary material for: Soil bacterial diversity is positively associated with air temperature in the maritime Antarctic
Source: Sci Rep. 2019 Feb 25;9:2686. doi: 10.1038/s41598-019-39521-7 (PMC6389919; doi:10.1038/s41598-019-39521-7)
Supplement: Supplementary file 1 — Supplementary Information [file 41598_2019_39521_MOESM1_ESM.pdf]

Supplementary information:

Soil bacterial diversity is positively associated with air temperature in the maritime Antarctic

Paul G. Dennis[1*], Kevin K. Newsham[2], Steven P. Rushton[3], Anthony G. O'Donnell[4], David W. Hopkins[5]

[1]*School of Earth and Environmental Sciences, The University of Queensland, Brisbane, QLD 4072, Australia;* [2]*NERC British Antarctic Survey, Madingley Road, Cambridge, CB3 0ET, UK;* [3]*School of Biology, Newcastle University, Newcastle upon Tyne, NE1 7RU, UK;* [4]*University of Western Australia, 35 Stirling Highway, Crawley, WA 6009, Australia;* [5]*Scotland's Rural College, Peter Wilson Building, West Mains Road, Edinburgh, EH9 3JG, UK.* *email: p.dennis@uq.edu.au

Contents:

Table S1 Site locations and associated data……………………………….……………...…..2

Table S2 Pearson's correlations between latitude and environmental parameters…………....3

Fig. S1 Phylogenetic neighbourhood of dominant OTUs from the Acidobacteria…………....4

Fig. S2 Phylogenetic neighbourhood of dominant OTUs from the Bacteroidetes…………....5

Fig. S3 Phylogenetic neighbourhood of dominant OTUs from the Cyanobacteria………..…6

Fig. S4 Phylogenetic neighbourhood of dominant OTUs from the Gemmatimonadetes……..7

Fig. S5 Phylogenetic neighbourhood of dominant OTUs from the Proteobacteria………..…8

Fig. S6 Positive correlation between air and soil temperatures at Mars Oasis ……………..…9

Fig. S7 OTUs associated with soil Mg concentration and moisture content…………….…..10

Table S1 Site names, locations, MASAT, altitudes and alpha diversity metrics

	Sampling site	Latitude and longitude	MASAT (°C)	Altitude (metres a.s.l.)	Soil pH value	Numbers of OTUs Observed*	Numbers of OTUs Predicted (Chao1)*	Faith's Phylogenetic Diversity Index*
1	Mars Oasis, Alexander Island	71.878° S, 68.248° W	-11.17	15	7.56	934	1999	39.6
2	Mars Oasis, Alexander Island	71.878° S, 68.248° W	-11.17	15	7.02	1234	2911	51.2
3	Mars Oasis, Alexander Island	71.878° S, 68.248° W	-11.17	15	7.46	1037	2328	39.4
4	Mars Oasis, Alexander Island	71.878° S, 68.248° W	-11.17	15	7.21	1007	2434	39.7
5	Mount Holt, Alexander Island	69.408° S, 71.665° W	-7.41	70	6.52	1366	3185	50.2
6	Hopkins Ridge, Alexander Island	69.366° S, 71.842° W	-7.41	66	6.23	1063	2454	45.5
7	Hopkins Ridge, Alexander Island	69.366° S, 71.844° W	-7.41	62	5.43	1771	4710	60.5
8	Hopkins Ridge, Alexander Island	69.367° S, 71.844° W	-7.41	70	6.55	1032	2029	47.5
9	Jenny Island	67.731° S, 68.365° W	-5.12	12	6.29	1363	3018	62.0
10	Jenny Island	67.731° S, 68.365° W	-5.12	12	6.29	1237	2870	48.6
11	Lagoon Island	67.594° S, 68.247° W	-5.12	20	5.38	1540	3673	60.7
12	Rothera Point, Adelaide island	67.568° S, 68.114° W	-6.70	6	6.62	805	1704	35.9
13	Blaiklock Island	67.543° S, 67.198° W	-8.50	7	6.78	1382	2964	49.6
14	Detaille Island	66.790° S, 66.869° W	-6.26	21	6.70	1636	3554	71.8
15	Detaille Island	66.790° S, 66.869° W	-6.26	26	6.41	2004	4852	79.7
16	Cape Evenson, Antarctic Peninsula	66.145° S, 65.717° W	-7.45	59	5.26	1527	3696	58.5
17	Cape Evenson, Antarctic Peninsula	66.145° S, 65.723° W	-7.45	31	5.66	1651	4007	66.8
18	Yelcho Station (CHL), Wiencke Island	64.894° S, 63.553° W	-5.11	6	7.72	1255	3261	53.8
19	Yelcho Station (CHL), Wiencke Island	64.894° S, 63.552° W	-5.11	6	6.48	1366	3178	59.1
20	Yelcho Station (CHL), Wiencke Island	64.837° S, 63.516° W	-5.11	2	5.11	1207	2882	47.6
21	Port Lockroy, Goudier Island	64.817° S, 63.483° W	-5.11	10	7.45	1595	3717	62.2
22	Marambio Station (ARG), Seymour Island	64.236° S, 56.626° W	-8.28	160	7.76	1580	3897	54.1
23	Alectoria Island	63.977° S, 58.640° W	-7.47	50	6.36	1494	3132	67.1
24	Spert Island	63.845° S, 60.951° W	-5.10	102	5.69	1516	3432	63.2
25	Spert Island	63.844° S, 60.945° W	-5.10	92	6.21	1915	5551	65.7
26	Newsham Nunatak	63.560° S, 57.825° W	-7.16	612	5.55	1083	2340	37.8
27	Newsham Nunatak	63.559° S, 57.824° W	-7.16	617	6.67	1617	3412	75.3
28	Whalers Bay, Deception Island	62.977° S, 60.552° W	-2.50	91	6.74	1141	2872	48.2
29	Whalers Bay, Deception Island	62.976° S, 60.557° W	-2.50	14	6.66	1240	3060	52.7
30	South Beaches, Byers Peninsula, Livingston Island	62.655° S, 61.090° W	-1.54	64	7.31	1579	3589	58.3
31	South Beaches, Byers Peninsula, Livingston Island	62.653° S, 61.092° W	-1.54	75	7.24	1652	3745	65.0
32	Edwards Point, Robert Island	62.460° S, 59.509° W	-3.05	26	5.49	1335	3038	44.0
33	Keller Peninsula, King George Island	62.086° S, 58.399° W	-2.95	65	6.81	1814	4132	67.9
34	Wynn Knolls, Signy Island	60.701° S, 45.662° W	-4.07	199	7.37	2167	5607	75.5
35	Wynn Knolls, Signy Island	60.701° S, 45.662° W	-4.07	199	6.93	1956	4379	73.7
36	Wynn Knolls, Signy Island	60.701° S, 45.662° W	-4.07	199	7.70	1988	4614	75.0
37	Wynn Knolls, Signy Island	60.701° S, 45.662° W	-4.07	199	7.46	1975	4664	73.8
38	Wynn Knolls, Signy Island	60.701° S, 45.662° W	-4.07	199	7.98	1841	4574	68.5
39	Wynn Knolls, Signy Island	60.701° S, 45.662° W	-4.07	199	7.23	1954	4092	79.5
40	Wynn Knolls, Signy Island	60.701° S, 45.662° W	-4.07	199	7.96	1868	3964	73.3

*Data are based on sequences clustered at 97% similarity and rarefied to 5,700 reads per soil. Abbreviations: MASAT, mean annual surface air temperature; OTUs, operational taxonomic units; CHL, Chile; ARG, Argentina

Table S2 Data from Pearson's correlations between latitude and environmental parameters

Parameter	r^2 value (%)	*P* value
MASAT* (° C)	65.0	<0.001***
Altitude (metres a.s.l.)	9.7	0.051
pH value	4.3	0.197
Electrical conductivity (μS cm^{-1})	0.0	0.957
Organic C concentration (mg kg^{-1} dry soil)	3.1	0.275
N concentration (mg kg^{-1} dry soil)	2.5	0.333
C:N ratio	40.7	<0.001***
Ca concentration (mg kg^{-1} dry soil)	2.3	0.357
Cu concentration (mg kg^{-1} dry soil)	0.2	0.807
Fe concentration (mg kg^{-1} dry soil)	0.9	0.565
K concentration (mg kg^{-1} dry soil)	1.9	0.392
Mg concentration (mg kg^{-1} dry soil)	26.7	<0.001***
Mn concentration (mg kg^{-1} dry soil)	1.0	0.537
P concentration (mg kg^{-1} dry soil)	1.7	0.421
Zn concentration (mg kg^{-1} dry soil)	3.3	0.258
Dissolved organic C concentration (mg kg^{-1} dry soil)	4.3	0.201
Dissolved NO_3^--N/NO_2^--N concentration (mg kg^{-1} dry soil)	0.5	0.653
Dissolved NH_4^+-N concentration (mg kg^{-1} dry soil)	0.6	0.646
Dissolved SO_4^{2-} concentration (mg kg^{-1} dry soil)	0.0	0.938
Dissolved PO_4^{3-} concentration (mg kg^{-1} dry soil)	1.2	0.500
Dissolved Cl^- concentration (mg kg^{-1} dry soil)	0.0	0.929
Moisture concentration (%)	0.1	0.881

Abbreviation: MASAT, mean annual surface air temperature

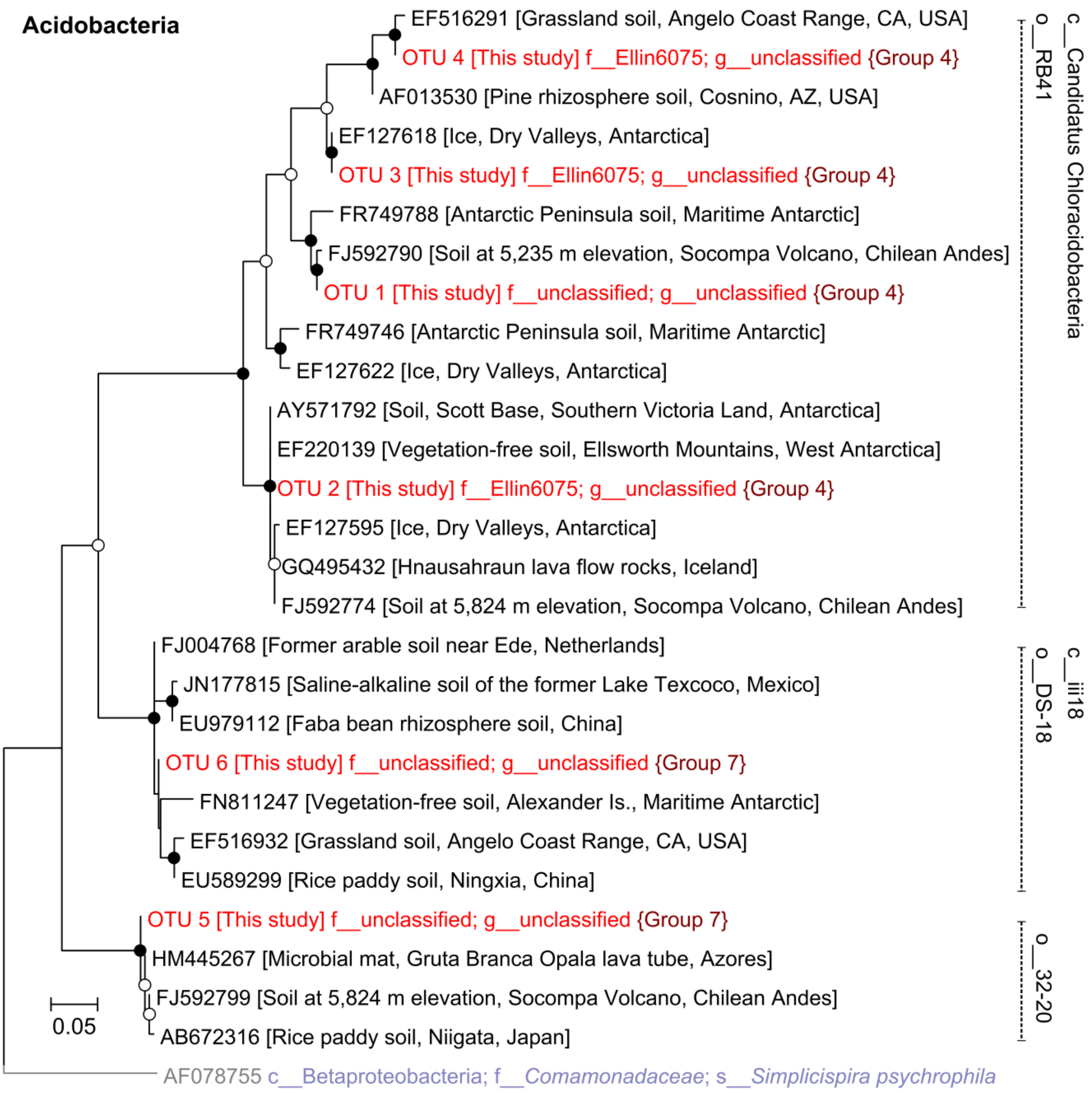

Fig. S1 Phylogenetic neighbourhood of dominant OTUs from the Acidobacteria. The OTUs from this study are shown in red with similar sequences from other studies shown in black. The percentage of trees from 1000 bootstraps in which the associated taxa clustered together is shown next to the branches, where open circles represent 50-74% and closed circles represent 75%-100%. The tree is drawn to scale, with branch lengths measured in the number of substitutions per site.

Bacteroidetes

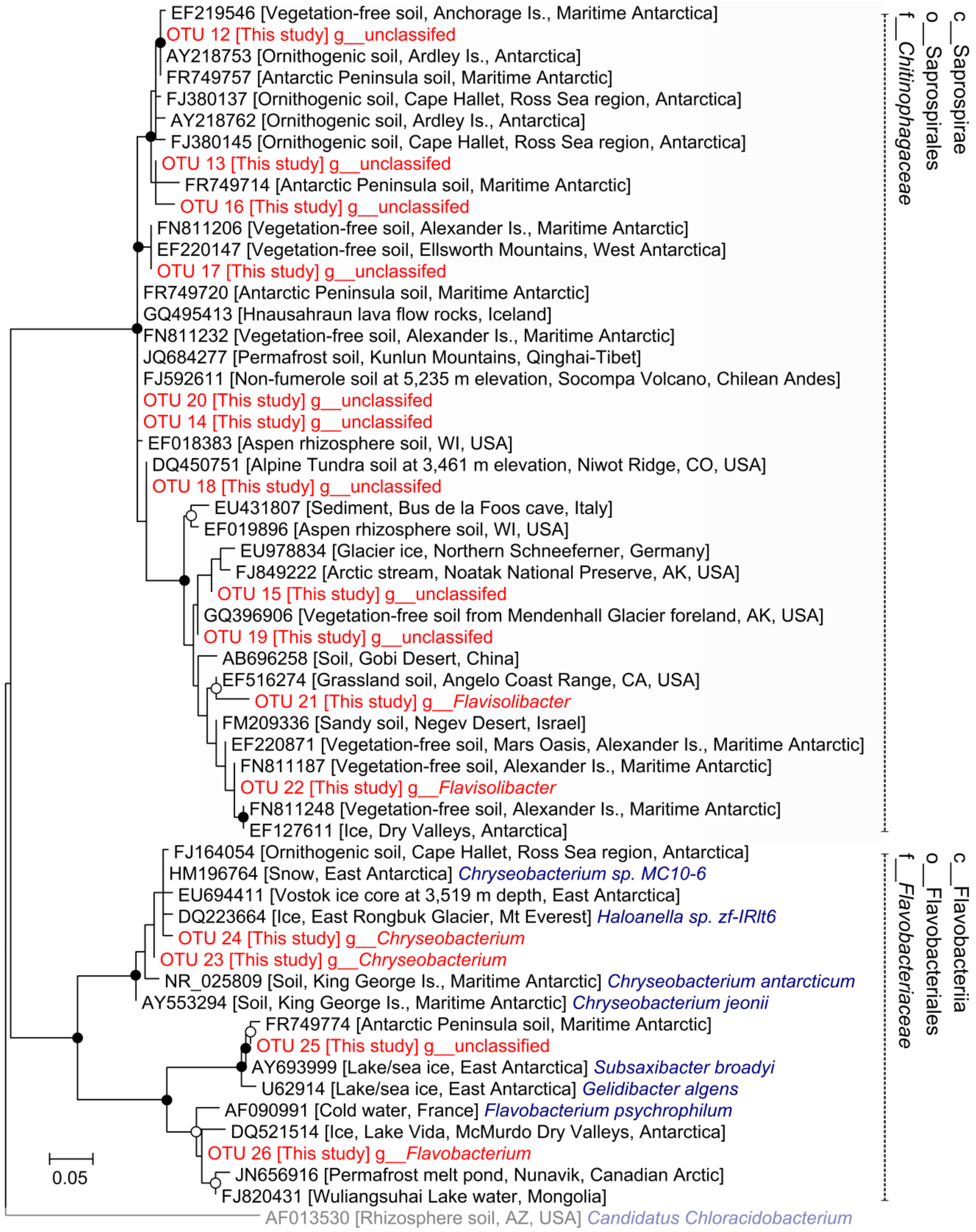

Fig. S2 Phylogenetic neighbourhood of dominant Bacteroidetes

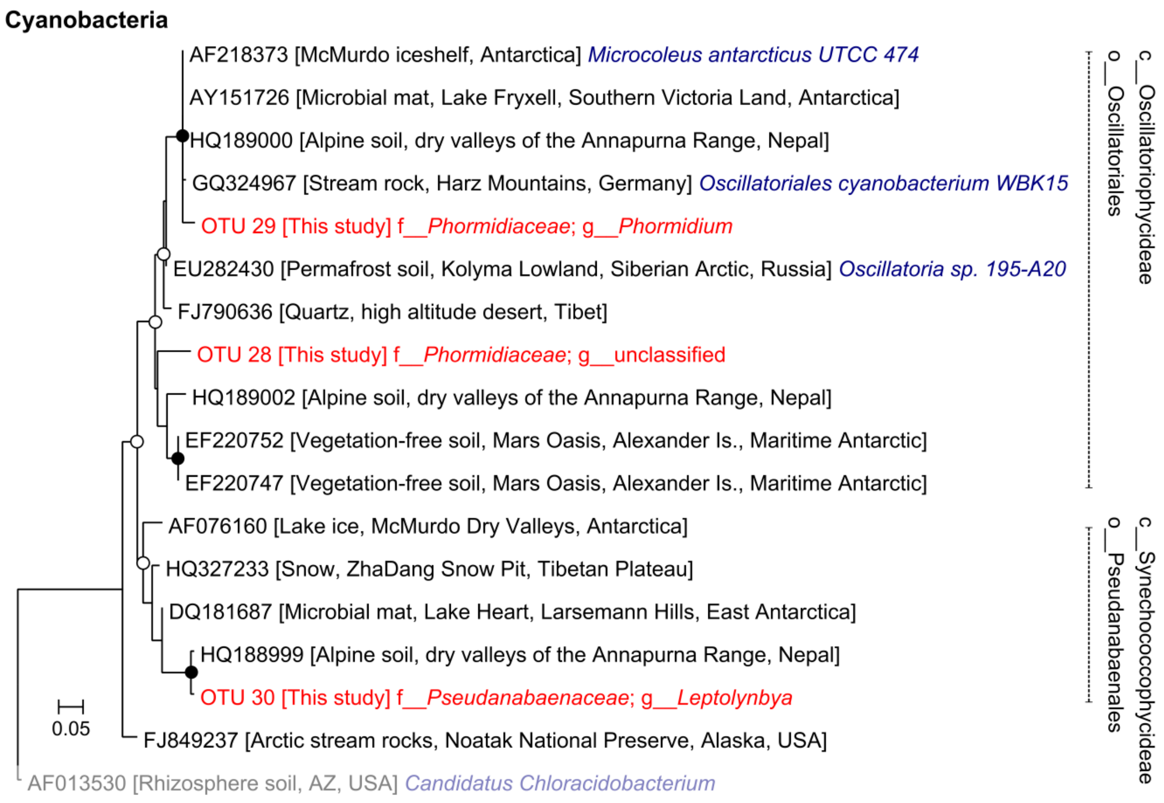

Fig. S3 Phylogenetic neighbourhood of dominant Cyanobacteria

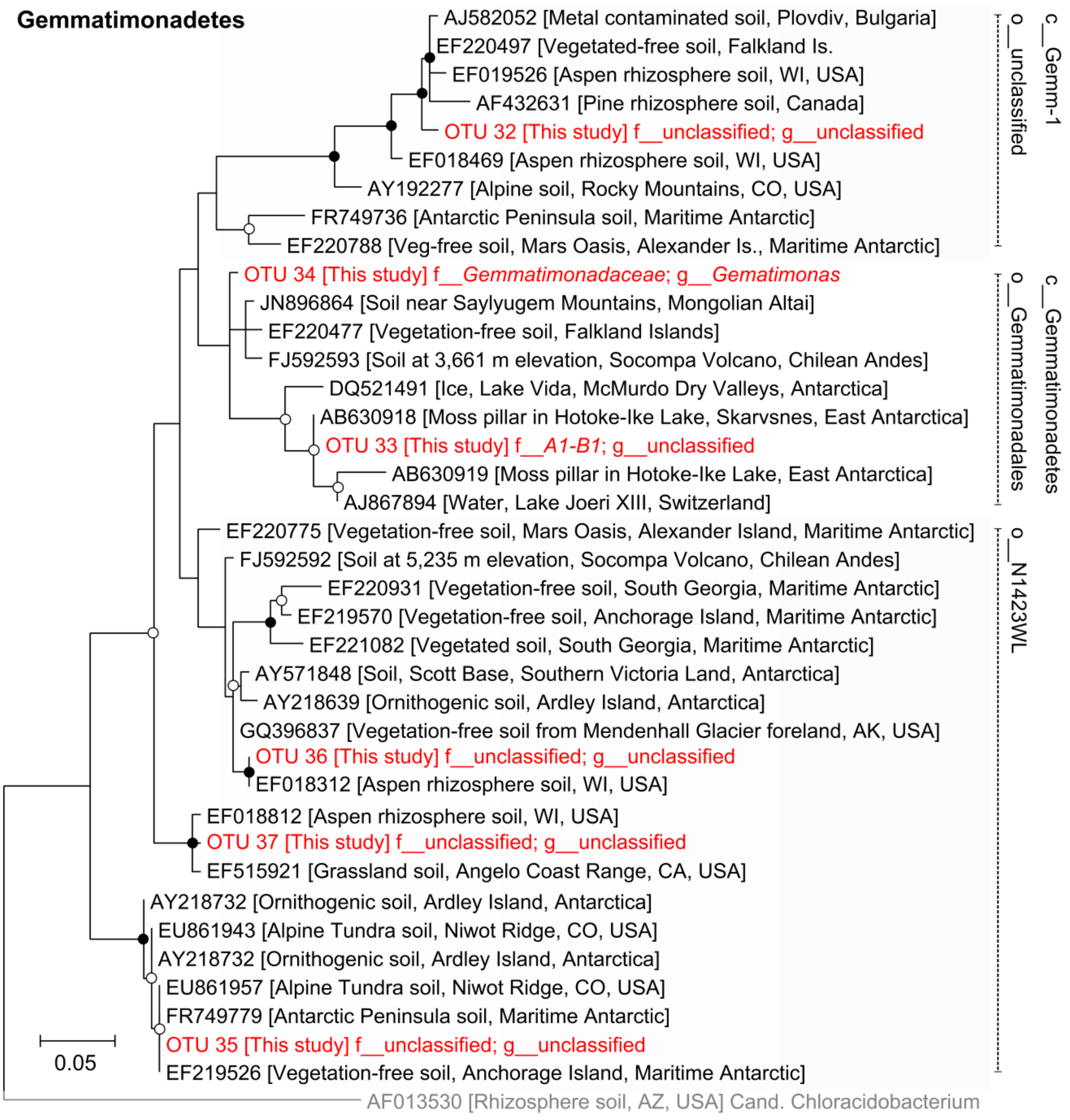

Fig. S4 Phylogenetic neighbourhood of dominant Gemmatimonadetes

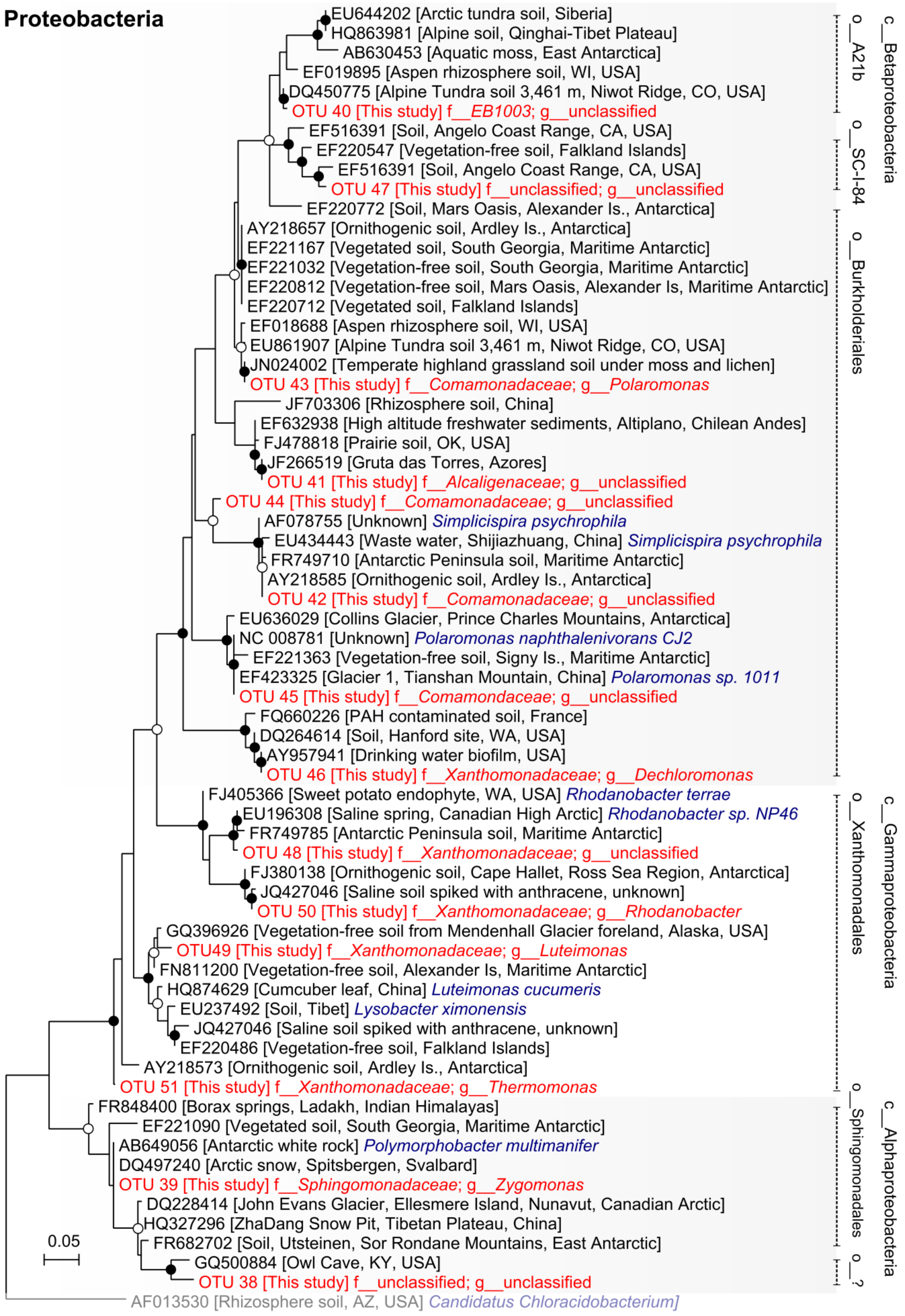

Fig. S5 Phylogenetic neighbourhood of dominant Proteobacteria

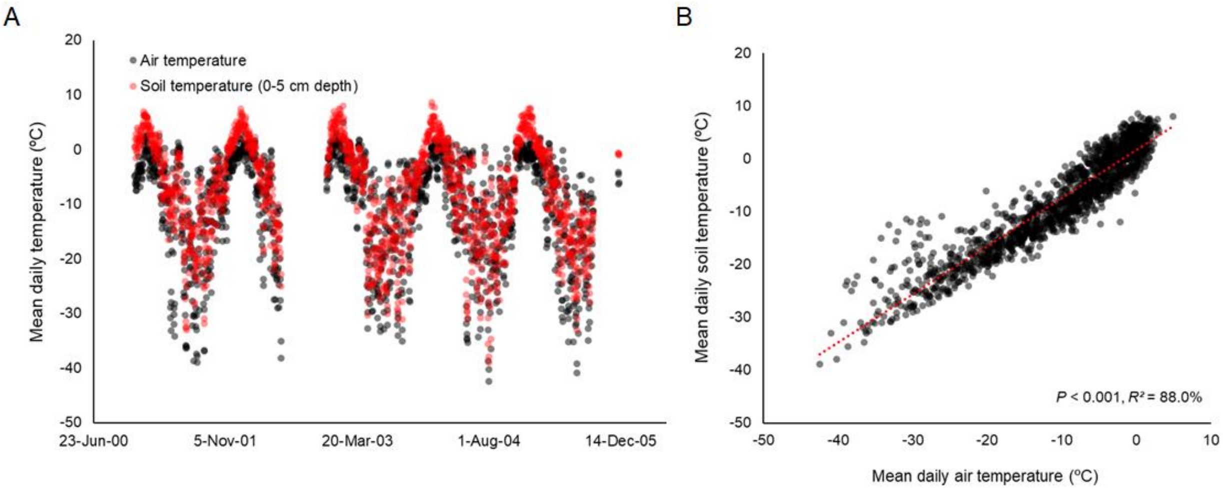

Fig. S6 (A) Mean daily air and soil temperatures from Mars Oasis for the period November 2000 until December 2005. Air temperatures were measured at a distance of one metre from the soil surface. Soil temperatures were measured at 0-5 cm depth. (B) A scatterplot of the air and soil temperatures shown in panel A with a line of best fit and the P and R^2 values from a linear regression model. Data were accessed from the UK Polar Data Centre, Antarctic Microclimate Data (GB/NERC/BAS/AEDC/00002) by contacting UK PDC at polardatacentre@bas.ac.uk [accessed: 16/01/2019].

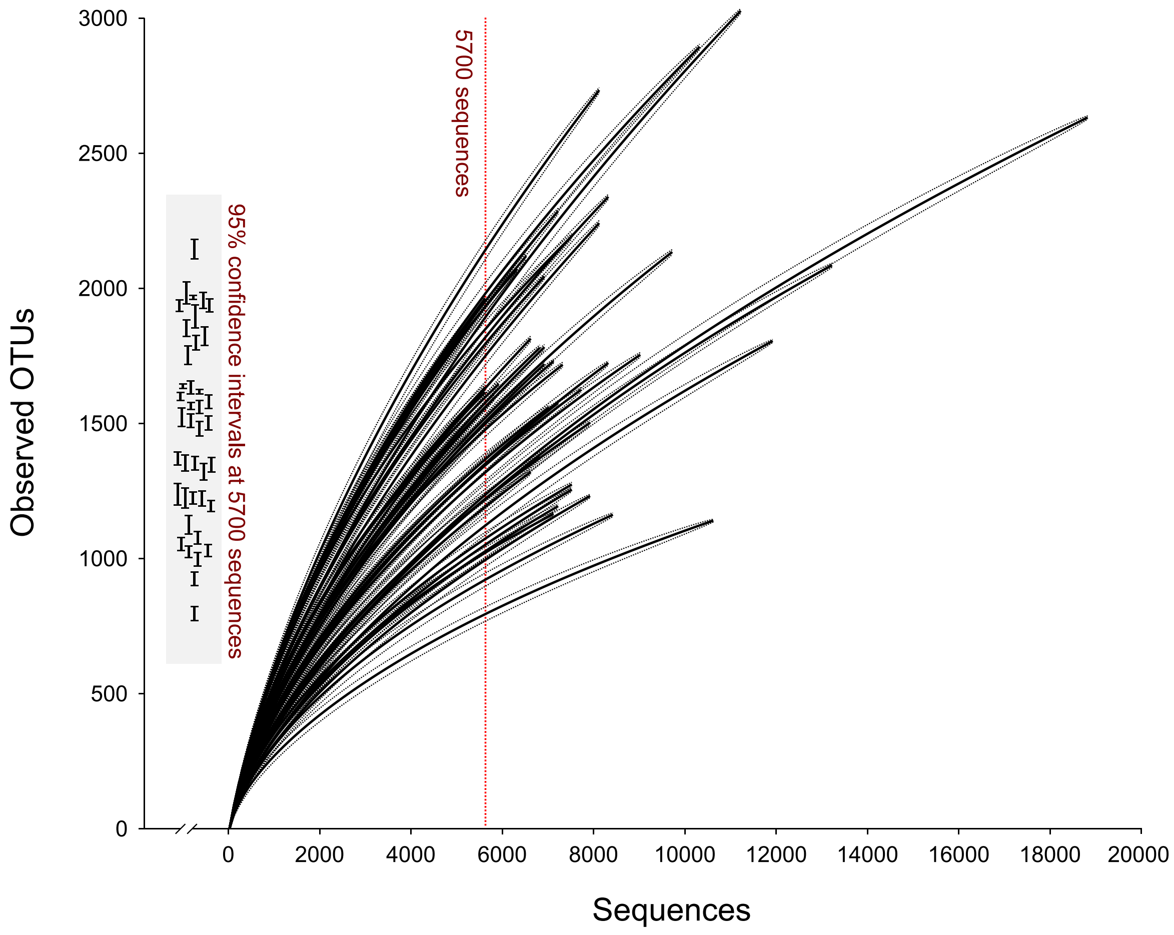

Fig. S7 Rarefaction curves representing the expected number of bacterial taxa for a given number of sequences per sample. The red dotted line marks 5,700 sequences per sample, which was the rarefaction level selected for all comparisons of diversity in this study. The dotted lines represent the 95% confidence intervals associated with each sample. To the left of the curves, the insert shows 95% confidence intervals for each sample at 5,700 sequences per sample. The values in the insert align with the y axis and are spaced arbitrarily on the x axis to avoid them overlapping one another.
